# Reversible contrast-induced encephalopathy after coil embolization of epistaxis

**DOI:** 10.5935/0103-507X.20210043

**Published:** 2021

**Authors:** Guilherme Menezes Mescolotte, Fernando Rodrigues da Silva, Susana Afonso, Jaime Pamplona, Rui Moreno

**Affiliations:** 1 Universidade Estadual de Campinas - Campinas (SP), Brazil.; 2 Neurocritical and Trauma Intensive Care Unit, Hospital de São José, Centro Hospitalar Universitário de Lisboa Central - Lisboa, Portugal.; 3 Neuroradiology Service, Hospital de São José, Centro Hospitalar Universitário de Lisboa Central - Lisboa, Portugal.

**Keywords:** Brain diseases/chemically induced, Contrast media/adverse effects, Epistaxis, Pregnancy complications, Encefalopatias/induzido quimicamente, Meios de contraste/efeitos adversos, Epistaxe, Complicações na gravidez

## Abstract

A 37-year-old woman (35 weeks pregnant) was admitted to a local hospital due to severe epistaxis resulting in shock and the need for emergency cesarean section. After failure to tamponade the bleeding, angiographic treatment was provided. After the procedure, she was admitted to the neurocritical intensive care unit and was confused and agitated, requiring sedation and endotracheal intubation. In the intensive care unit, diagnostic investigations included brain magnetic resonance imaging, lumbar puncture with viral panel, electroencephalogram, tests for autoimmunity, and hydroelectrolytic and metabolic evaluations. Magnetic resonance imaging showed a puntiform restricted diffusion area on the left corona radiata on diffusion weighted imaging and mild cortical posterior edema (without restricted diffusion), and an electroencephalogram showed moderate diffuse slow activity and fronto-temporal slow activity of the left hemisphere with associated scarce paroxysmal components. The other exams did not show any relevant alterations. Due to the temporal relationship, the clinical history and the magnetic resonance imaging results, a diagnosis of contrast-induced encephalopathy was made. After 2 days in the intensive care unit, sedation was withdrawn, the patient was extubated, and total neurological recovery was verified within the next 24 hours.

## Abstract (pt)

Uma mulher com 37 anos de idade, gestante de 35 semanas, foi admitida em um hospital local em razão de epistaxe grave, que resultou em choque e em necessidade de realização de cesárea emergencial. Após falha do tamponamento para controlar a hemorragia, decidiu-se por tratamento angiográfico. Após o procedimento, ela foi admitida à unidade de terapia intensiva neurocrítica, encontrando-se confusa e agitada, com necessidade de sedação e intubação orotraqueal. Na unidade de terapia intensiva, as investigações incluíram exames de ressonância magnética, punção lombar com painel viral, eletroencefalograma, testes para autoimunidade e avaliações hidroeletrolítica e metabólica. O exame de ressonância magnética mostrou área puntiforme restrita na corona radiata esquerda nas sequências de imagens pesadas em difusão, além de leve edema cortical posterior (sem restrição à difusão), e o eletroencefalograma mostrou atividade lenta difusa moderada, atividade frontoparietal lenta e escassos componentes paroxísticos associados no hemisfério esquerdo. Outros exames não mostraram alterações relevantes. Por causa da relação temporal e da história clínica, assim como imagens de ressonância magnética, formulou-se o diagnóstico de encefalopatia induzida por contraste. A sedação foi retirada após 2 dias na unidade de terapia intensiva, e a paciente foi extubada, verificando-se completa recuperação neurológica dentro das 24 horas seguintes.

## INTRODUCTION

Contrast media has revolutionized medicine. Through the use of contrast, we highlight the images obtained by X-ray emission devices or another types of imaging diagnostic methods, and thus increase the accuracy of the tests.^(1)^ The first use of intravenous contrast was performed in 1896 by Haschek and Lindenthal, who injected mercury into an amputated arm.^(2)^ With the diffusion of contrast angiographic techniques in 1950, Mani and Eisenberg reported a rate of 0.3% to 2.5% neurological complications related to contrast infusion during angiographies.^(3)^

Currently, with improved contrast techniques and the use of less toxic contrast media, contrast-induced encephalopathy (CIE) is extremely rare. Although the symptoms in most cases are transient, they are debilitating and need to be well evaluated because their differential diagnosis can include life-threatening conditions,^(4)^ and in very rare situations have led to persistent deficits or even death.^(5)^

In the hours following any type of contrast infusion, we can potentially see signs of encephalopathy or focal deficits (e.g., parkinsonism, hemianopia, cortical blindness, hemiparesis, hemianesthesia).^(6)^ The pathophysiology of these manifestations is not well understood, but it is believed that a breakdown of the blood-brain barrier, driven by a direct excitatory effect of contrast media, causes neuronal toxicity that manifests as encephalopathy, even with the use of low osmolality and nonionic organic contrast media.^(6)^

## CASE DESCRIPTION

A 37-year-old Caucasian woman who was 35 weeks pregnant with a history of 2 previous spontaneous abortions experienced a sudden, spontaneous, and very heavy epistaxis from the left nostril. Conducted to the emergency department of the local hospital, she presented hemodynamic instability and fetal monitorization compatible with severe fetal distress. She underwent an emergency cesarean section preceded by a focal convulsion upon the induction of anesthesia (attributed to induction of anesthesia in the context of severe hypovolemic anemic shock leading to cerebral hypoperfusion). The epistaxis continued and she received 5 units of red blood cells and 1 unit of fresh frozen plasma over 2 days. Because of failure to control the nasal bleeding with a local hemostatic balloon, she was transferred to a tertiary hospital for angiography with eventual embolization. During this period, she did not present any changes in her mental state or hemodynamic stability, and the local hemostatic balloon in the left nostril was kept in place.

The first angiography was performed using 50mL of low osmolality and nonionic organic contrast media administered in the internal and external carotid arteries. There was an abnormal vascular blush in the left nasal hemifossa dependent on branches of the sphenopalatine artery, and embolization was performed. However, the epistaxis returned shortly after, and a second angiography was performed 2 hours later, excluding the presence of any remaining vascular blush and confirming the overall success of the prior embolization. A total of 90mL of nonionic isosmolar contrast was used in both angiographies. Local tamponing was kept in the left nostril.

Immediately after the second angiography, during the postanaesthetic recovery, the patient started to be disoriented, with sleepy speech but no formal visual complaints or motor deficits. Within 2 hours of the second procedure, she remained alert but cried continuously, developed hyperventilation, was not able to follow orders, directed her gaze but could not fix it and presented repetitive speech without intelligibility or the ability to make sentences. She was hemodynamically stable, afebrile and without any meningeal signs. The patient was transferred to the intensive care unit (ICU) due to her clinical evolution and the eventual need for airway protection in the context of an acute confusional state of unknown cause.

Given the patient’s agitation and noncooperation, it was not possible to properly evaluate her language and content of thought or humor, and it was not possible at that time to exclude the possibility of aphasia. A formal psychiatric evaluation, performed within a few hours of presentation, ruled out any major psychiatric illness, as well as the more common peripartum psychosis (PPP). There were no motor deficits, cranial nerve changes, or other focal signs suggesting extensive focal neurological injury. Given the acute onset, brain magnetic resonance imaging (MRI) was performed to evaluate possible complications of angiography, and a lumbar puncture was performed to exclude an infectious or inflammatory disease of the central nervous system. An electroencephalogram was also performed.

**Figure 1:**
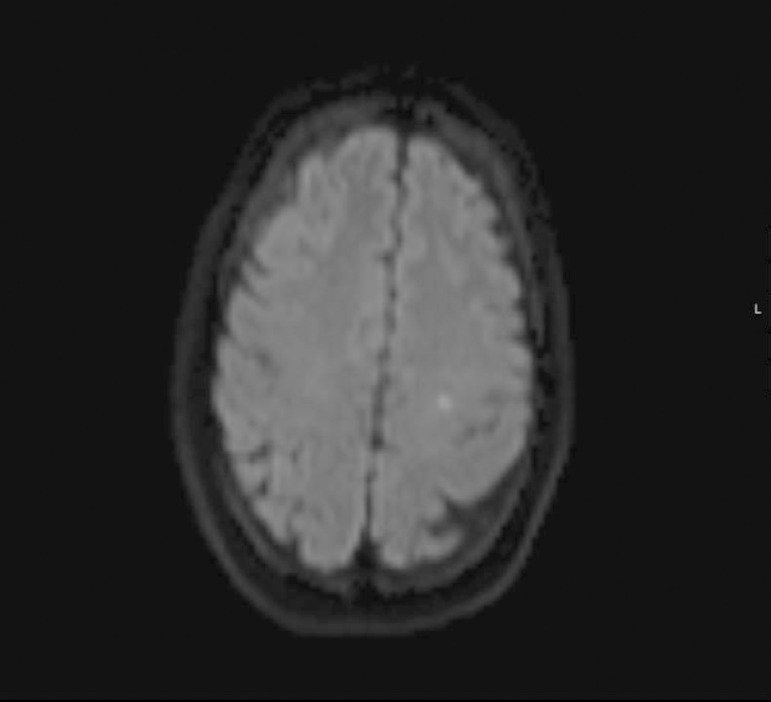
Magnetic resonance imaging (axial diffusion weighted imaging) showing a small lesion with restricted diffusion on the left corona radiata.

**Figure 2:**
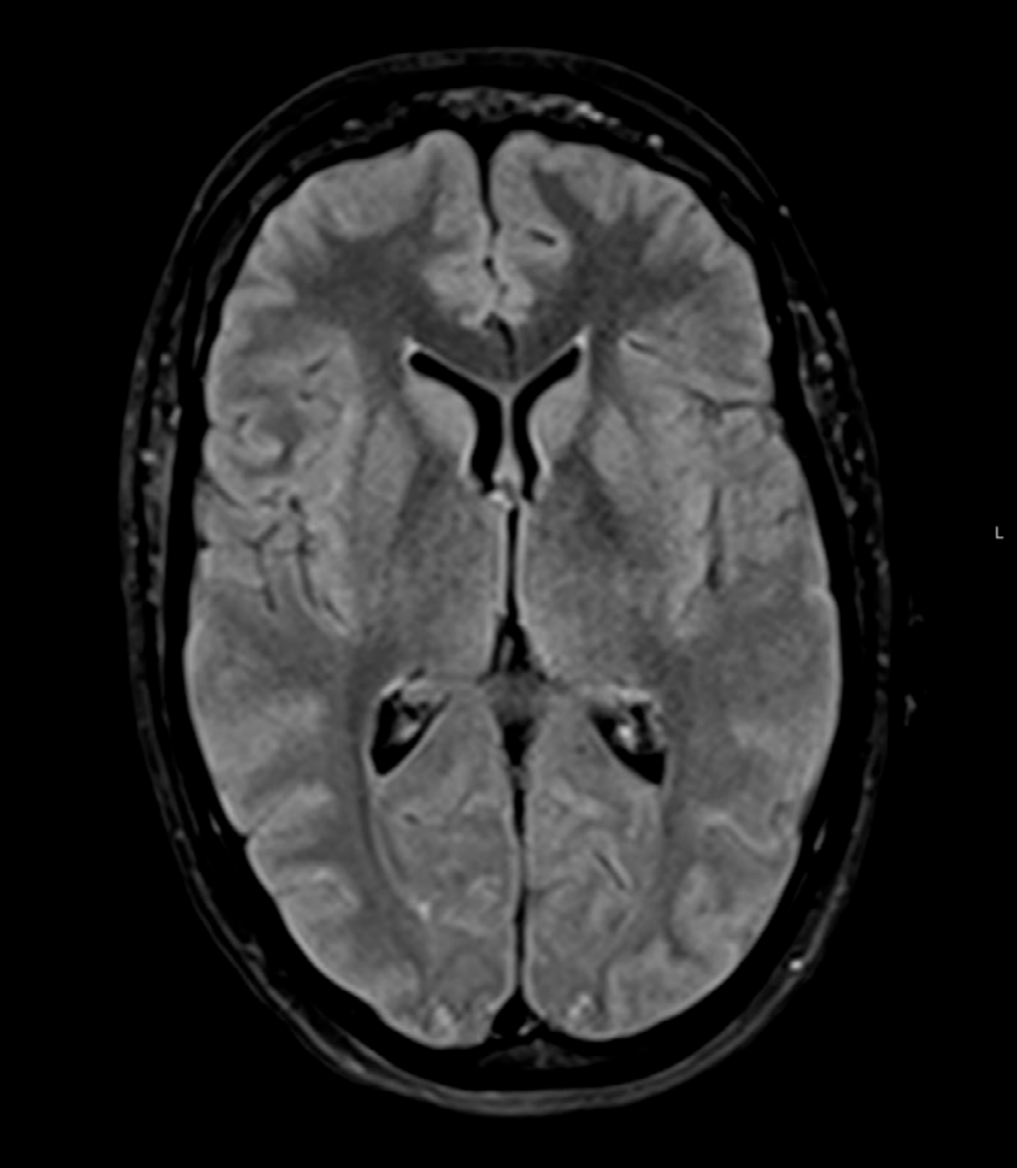
Magnetic resonance imaging (axial fluid-attenuated inversion recovery) showing smooth swelling of the posterior temporo-occipito-parietal cortex, bilaterally, with discrete hypersignal.

Magnetic resonance imaging revealed a small focus of hypersignal on the diffusion weighted imaging at the level of the left corona radiata white matter (Figure 1), with hyposignal on the apparent diffusion coefficient map, with a discrete hypersignal in the fluid-attenuated inversion recovery. There was also smooth swelling of the posterior temporo-occipito-parietal cortex bilaterally, with a discrete hypersignal on the fluid-attenuated inversion recovery (Figure 2) but without restricted diffusion, suggesting vasogenic edema. Her cerebrospinal fluid was crystal clear, with a normal opening pressure, and presented four cells, a glucose of 72mg/dL, proteins 53mg/dL and a lactate dehydrogenase (LDH) < 30UI/L. Polymerase chain reaction was negative for an extensive panel of neurotropic viruses; all bacterial cultures were negative. There were no electrolytic disturbances, and systemic tests for autoimmunity were negative.

An electroencephalogram showed predominant theta activity in the posterior projection, beta activity in the anterior and theta-delta activity infrequent in the frontotemporal region, and a left predominance, sometimes with a rhythmic morphology followed by base suppression. The traces revealed diffuse slowing of base electrogenesis to a moderate degree. There was fronto-temporal slow activity of the left hemisphere associated with a scarce paroxysmal component but it was mild and noncontinuous.

Sedoanalgesia and mechanical ventilation were maintained for 2 days, being withdrawn only after stabilization of the clinical picture and reviewing the results of the MRI and electroencephalogram to exclude further brain damage that could need a different approach.

Extubated, she maintained a very poor vocabulary (with a maximum of 40 spoken words) but with no focal deficits. In less than 24 hours, a progressive improvement of her speech and reasoning content was observed, without other changes in the examination. Her renal function was normal throughout.

**Figure 3:**
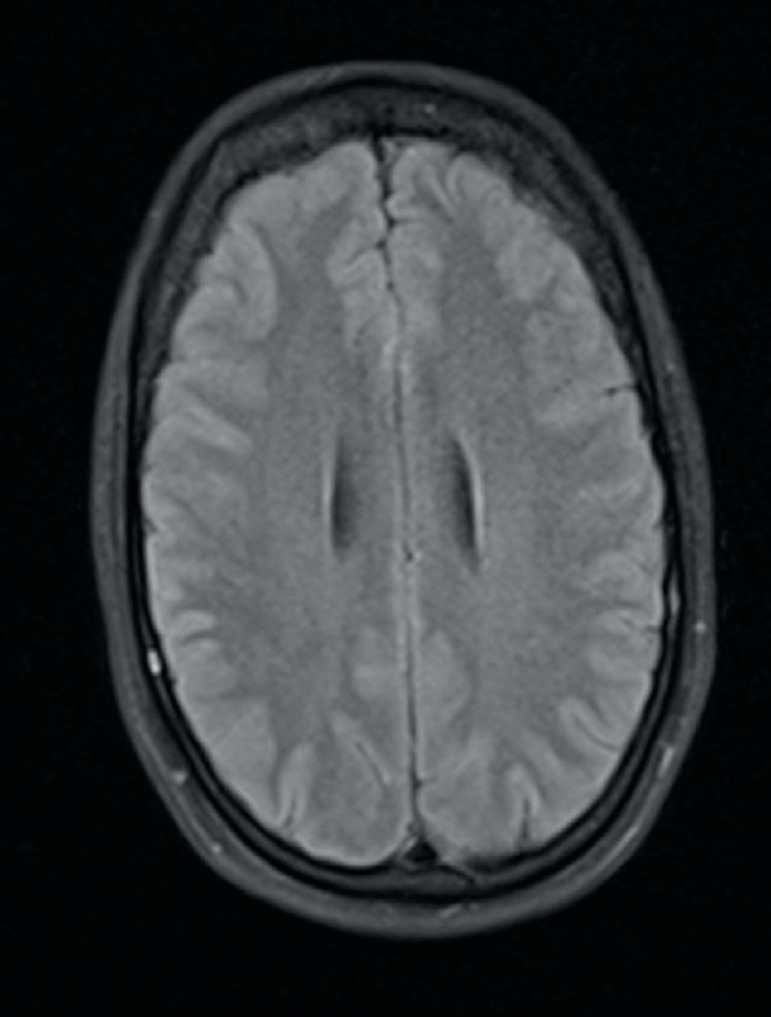
Magnetic resonance imaging (axial fluid-attenuated inversion recovery), with complete resolution of the posterior cortical edema.

A second MRI was performed 48 hours after the first, showing the presence of a few bilateral upper cortical/subcortical millimetric restricted diffusion lesions, probably related to the previous angiography. There was a complete resolution of her posterior cortical edema (Figure 3). The rest of her brain parenchyma did not presented other relevant changes.

Clinically, the patient progressed very well, with quick recovery of speech and all neurological changes. Tamponing was withdrawn on the third day, and the patient was discharged home 5 days after the C-section.

## DISCUSSION

In the face of the initial clinical picture, the first diagnosis made by the attending obstetrician was eclampsia. Eclampsia is defined as coma or seizures in a pregnant woman with preeclampsia (hypertension plus target organ damage) or gestational hypertension. The eclampsia hypothesis was promptly excluded because the patient only presented seizures in the context of induction of anesthesia and severe hypotension in a patient with severe hypovolemic shock (hemoglobin 7g/L after 4 units of packed red blood cells). Additionally, in her obstetric past-including the present pregnancy-she did not present hypertension, proteinuria or new-onset cerebral and visual disturbances.^(7,8)^

Mental confusion with restlessness without focal deficit 4 hours after the first angiography initially raised the hypothesis of encephalopathy of unknown cause, acute PPP, or ischemic injury. Encephalopathy is an alteration of the mental state caused by diffuse brain injury. In pregnancy, many disorders can present as encephalopathy, such as stroke, intracranial venous sinus thrombosis, demyelinating diseases, vasculitis, metabolic alterations, encephalitis and posterior reversible encephalopathy syndrome (PRES). A psychiatric disease was considered very improbable by the attending psychiatrist because PPP usually presents between 1 and 4 weeks postpartum and is usually more insidious. The magnetic resonance findings ruled out ischemic lesions and PPP. By exclusion, the puntiform restricted diffusion lesion on the left corona radiata was attributed to the angiographic procedure but it could not *per se* explain the full clinical picture.^(9)^

Given this, the main differential diagnoses for the bilateral vasogenic edema of the posterior temporo-occipito-parietal cortex were CIE and posterior PRES. For the PRES diagnosis, there was a lack of some important features, such as a history of preeclampsia or severe hypertension prior to the onset of clinical symptoms, subcortical edema of the posterior region and microhemorrhagic lesions not seen on MRI.^(10)^

Thus, due to the temporal relationship between the angiographic procedures, the clinical symptoms and the cortical edematous changes in the posterior region (that spontaneously regressed after 48 hours), we made a definitive diagnosis of reversible CIE. Magnetic resonance imaging is an important tool in the differential diagnosis of CIE; basically, CIE is characterized by cortical and/or subcortical edema, more common in the posterior region, with spontaneous regression in most cases, with an epidemiological history of recent exposure to contrast. Other findings on MRI make the diagnostic hypothesis of CIE less likely.^(5)^

## CONCLUSION

The authors presented a case of reversible contrast-induced encephalopathy and would like to stress the need to consider this rare complication of its administration, even when using low osmolality and nonionic organic contrast media. Contrast media is a routine part of the approach to patients needing angiography for the diagnosis or control of arterial bleeding lesions, as well as in other clinical situations.

## References

[1] Christiansen C (2005). X-ray contrast media--an overview. Toxicology.

[2] Haschek E, Lindenthal OT (1896). A contribution to the practical use of the photography according to Röntgen. Wien Klin Wochenschr.

[3] Mani RL, Eisenberg RL (1978). Complications of catheter cerebral arteriography: analysis of 5,000 procedures. III. Assessment of arteries injected, contrast medium used, duration of procedure, and age of patient. AJR Am J Roentgenol.

[4] Yu J, Dangas G (2011). Commentary. New insights into the risk factors for contrast-induced encephalopathy. J Endovasc Ther.

[5] Leong S, Fanning NF (2012). Persistent neurological deficit from iodinated contrast encephalopathy following intracranial aneurysm coiling. A case report and review of the literature. Interv Neuroradiol.

[6] Chu YT, Lee KP, Chen CH, Sung PS, Lin YH, Lee CW (2020). Contrast-induced encephalopathy after endovascular thrombectomy for acute ischemic stroke. Stroke.

[7] Peraçoli JC, Borges VT, Ramos JG, Cavalli RC, Costa SH, Oliveira LG (2019). Pre-eclampsia/eclampsia. Rev Bras Ginecol Obstet.

[8] Vaughan CJ, Delanty N, Delanty N (2002). Pathophysiology of acute symptomatic seizures. Seizures: medical causes and management.

[9] Sit D, Rothschild AJ, Wisner KL (2006). A review of postpartum psychosis. J Womens Health (Larchmt).

[10] Hobson EV, Craven I, Blank SC (2012). Posterior reversible encephalopathy syndrome: a truly treatable neurologic illness. Perit Dial Int.

